# Strain-Reduced Micro-LEDs Grown Directly Using Partitioned Growth

**DOI:** 10.3389/fchem.2021.639023

**Published:** 2021-03-10

**Authors:** Shunpeng Lu, Yiping Zhang, Zi-Hui Zhang, Ping Chieh Tsai, Xueliang Zhang, Swee Tiam Tan, Hilmi Volkan Demir

**Affiliations:** ^1^ LUMINOUS! Center of Excellence for Semiconductor Lighting and Displays, The Photonics Institute, School of Electrical and Electronic Engineering, Nanyang Technological University, Singapore, Singapore; ^2^ Key Laboratory of Electronic Materials and Devices of Tianjin, School of Electronics and Information Engineering, Hebei University of Technology, Tianjin, China; ^3^ School of Energy and Chemical Engineering, Xiamen University Malaysia, Sepang, Malaysia; ^4^ School of Physics and Mathematical Sciences, Nanyang Technological University, Singapore, Singapore; ^5^ Department of Electrical and Electronic Engineering, Department of Physics, UNAM-Institute of Material Science and Nanotechnology, Bilkent University, Ankara, Turkey

**Keywords:** micro-LED, strain release, partitioned growth model, size effect, QCSE, Raman

## Abstract

Strain-reduced micro-LEDs in 50 μm × 50 μm, 100 μm × 100 μm, 200 μm × 200 μm, 500 μm × 500 μm, and 1,000 μm × 1,000 μm sizes were grown on a patterned c-plane sapphire substrate using partitioned growth with the metal-organic chemical-vapor deposition (MOCVD) technique. The size effect on the optical properties and the indium concentration for the quantum wells were studied experimentally. Here, we revealed that the optical properties can be improved by decreasing the chip size (from 1,000 to 100 µm), which can correspondingly reduce the in-plane compressive stress. However, when the chip size is further reduced to 50 μm × 50 μm, the benefit of strain release is overridden by additional defects induced by the higher indium incorporation in the quantum wells and the efficiency of the device decreases. The underlying mechanisms of the changing output power are uncovered based on different methods of characterization. This work shows the rules of thumb to achieve optimal power performance for strain-reduced micro-LEDs through the proposed partitioned growth process.

## Introduction

During the past few decades, InGaN/GaN based light-emitting diodes (LEDs) have been extensively studied to improve the external quantum efficiency (EQE) and optical output power (Shuji et al., 1991; Tan et al., 2012; Ji et al., 2013; Zhang et al., 2013). Owing to the high efficiency, long lifetime, and versatile packaging flexibility, III-nitride based LEDs have been widely used as the backlighting for smartphones and flat-panel displays, and are very promising for applications such as visible light communication and micro-displays (Choi et al., 2004a; McKendry et al., 2010; McKendry et al., 2012). All these applications require small LED chips, which have superior properties including high current density, high power density and high response speed. Thus, small LEDs that hold these properties are imperatively needed. To this end, micro-LEDs have been previously reported (Choi et al., 2003; Choi et al., 2004b; Poher et al., 2008; Lu et al., 2014). Micro-LEDs can deliver much higher current density and power density than general broad-area LEDs due to the reduced thermal mass and Joule heating (Lu et al., 2014). Nevertheless, to reduce the costs and satisfy the outdoor applications of micro-displays and visible light communication, the performance of micro-LEDs needs to be further improved. However, in all of these previous reports, the top-down approach based on dry etching and patterning micro-size mesas was used and the performance of the LED epitaxial wafers grown on the c-plane sapphire substrates is limited by the quantum confined Stark effect (QCSE) (Ryou et al., 2009). The strain induced large piezoelectric polarization fields contribute to the internal electric field reducing the oscillator strength and emission due to the QCSE (Tetsuya et al., 1997). To overcome this issue, numerous efforts have been made to release the strain. For example, nonpolar tetragonal LiAlO_2_ substrate was used to reduce the QCSE by Waltereit et al. (Waltereit et al., 2000). InGaN/GaN multiquantum well (MQW) nanorod arrays were implemented to improve the efficiency of LEDs by Kim et al. (Kim et al., 2004). Graphene-assisted growth was carried out to address the strain-induced problems in LEDs (Chen et al., 2018; Wang et al., 2020). Substrates such as semi-polar and non-polar bulk GaN were used to achieve high internal quantum efficiency (IQE) and low droop LEDs by Nakamura et al. (Arpan et al., 2005; Denbaars et al., 2013). However, these proposed methods come at high costs of substrates or nanoimprint lithography technology. On the other hand, it is deemed that if the micro-LEDs could be directly *in situ* grown in the metal-organic chemical-vapor deposition (MOCVD) chamber by adopting the partitioned growth process, the QCSE can be reduced in such partition-grown micro-LEDs (PG micro-LEDs). Furthermore, compared with typically fabricated micro-LEDs using ICP etching, there is no side-wall defects, and the efficiency will be even higher. Therefore, in this work, we propose and show a low-cost and effective way to release the strain for achieving high-efficiency strain-reduced micro-LEDs through partitioned growth.

In this work, strain-reduced micro-LEDs with various sizes of 50 μm × 50 μm, 100 μm × 100 μm, 200 μm × 200 μm, 500 μm × 500 μm, and 1,000 μm × 1,000 µm are demonstrated to be directly grown on patterned c-plane sapphire substrates by using our MOCVD system. To protect these PG micro-LEDs during the dicing process, a margin (10 µm) was included between two individual chip partitions. Here we systematically investigated the size effect on the strain and studied the electrical and optical properties for the proposed PG micro-LEDs which are critical to optimize the size for PG micro-LEDs.

## Materials and Methods

The LED epitaxial wafers were grown on c-plane single polished sapphire substrates by the MOCVD system. The sapphire substrate was first deposited with SiO_2_ (100 nm) by plasma enhanced chemical vapour deposition (PECVD), and the SiO_2_ layer was then patterned and etched with reactive-ion etching (RIE) to obtain square opening regions with different sizes. The margin width between two opening regions was set to 10 µm. Trimethylaluminum (TMAl), trimethylindium (TMIn), trimethylgallium (TMGa), and ammonia (NH_3_) were used as Al, In, Ga, and N precursors, respectively. The growth was initiated with a 3 µm thick unintentionally doped GaN, followed by a 5.5 µm thick Si-doped N-GaN (doping concentration ≈ 5 × 10^18^/cm^3^). Then, six pairs of In_0.15_Ga_0.85_N/GaN multiple quantum wells (MQWs) (thickness of 3 nm/12 nm) were grown. A Mg-doped Al_0.15_Ga_0.85_N electron blocking layer (EBL), with a thickness of 20 nm, was grown to reduce the electron overflow. Finally, a 200 nm thick Mg-doped GaN (with a free hole concentration of 3 × 10^17^/cm^3^) was grown as the hole source layer. In both of the EBL and the hole source layers, bis(cyclopentadienyl)magnesium (Cp2Mg) was used as the Mg precursor.

The images of different PG micro-LEDs were taken by a scanning electron microscopy (SEM) (JEOL JSM-5600LV) system. Micro-Raman spectra were also recorded using a spectrometer (Horiba JY-T64000) equipped with an excitation laser of 532 nm wavelength to reveal the strain level. Electroluminescence (EL) spectra and the optical output power were acquired by an Ocean Optics spectrometer (QE65000) attached to an integrating sphere. The micro-LEDs were fabricated into the same size (1 mm × 1 mm) using micro-fabrication technique. A LED tester (M2442S-9A Quatek Group) was used to measure the current-voltage characteristics of the resulting LED chips.

## Results and Discussion

**FIGURE 1:**
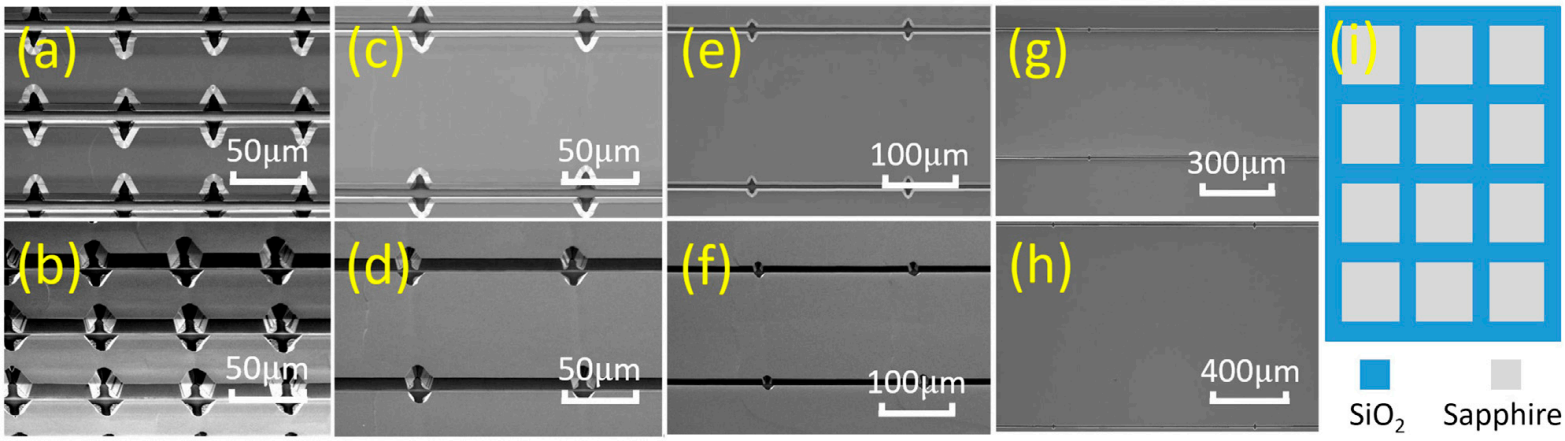
SEM images of the proposed PG micro-LEDs grown in different sizes: top-view images for **(A)** 50 μm × 50 μm, **(C)** 100 μm × 100 μm, **(E)** 200 μm × 200 μm, **(G)** 500 μm × 500 μm, and **(H)** 1,000 μm × 1,000 µm chips; 45°-tilted-view images for **(B)** 50 μm × 50 μm, **(D)** 100 μm × 100 μm, and **(F)** 200 μm × 200 µm chips. **(I)** The schematic diagram of the patterned substrate before growth with SiO_2_ on the sapphire substrate.

Figure 1 shows the SEM images for the PG micro-LEDs of different sizes and the pattern before growth. Here Figures 1A,C,E,G,H show the top-view images for the 50 μm × 50 μm, 100 μm × 100 μm, 200 μm × 200 μm, 500 μm × 500 μm, and 1,000 μm × 1,000 μm PG micro-LEDs, respectively. Figures 1B,D,F display the 45°-tilted-view images for the 50 μm × 50 μm, 100 μm × 100 μm, and 200 μm × 200 μm PG micro-LEDs, respectively. Figure 1I depicts the patterned sapphire substrate with SiO_2_ before the epitaxial layers growth. As the top-view and 45°-tilted-view images for the 500 μm × 500 µm and 1,000 μm × 1,000 µm sizes look similar, here we only show the top-view images for these two sizes. From these images, we can observe that smaller PG micro-LEDs have a larger ratio of opening area. Since the chip dimension for PG micro-LEDs is smaller than the anode, which is made of a 1 mm × 1 mm contact on the P-GaN surface, the active light emission area has to be taken into consideration. To facilitate the analysis, we used the square of the side length over the square of the side length plus half of the margin width (10 µm) as the ratio of active lighting area. For example, the ratio of active lighting area for the 50 μm × 50 µm device is 50 × 50 over (50 + 5) × (50 + 5). After calculation, we find that the ratios of active lighting area are 83, 91, 95, 98, and 99%, respectively, for the 50 μm × 50 μm, 100 μm × 100 μm, 200 μm × 200 μm, 500 μm × 500 μm, and 1,000 μm × 1,000 µm devices. From the calculation results, we can see that 50 μm × 50 μm PG micro-LEDs possess a much smaller ratio of the active light emission area than others.

**FIGURE 2:**
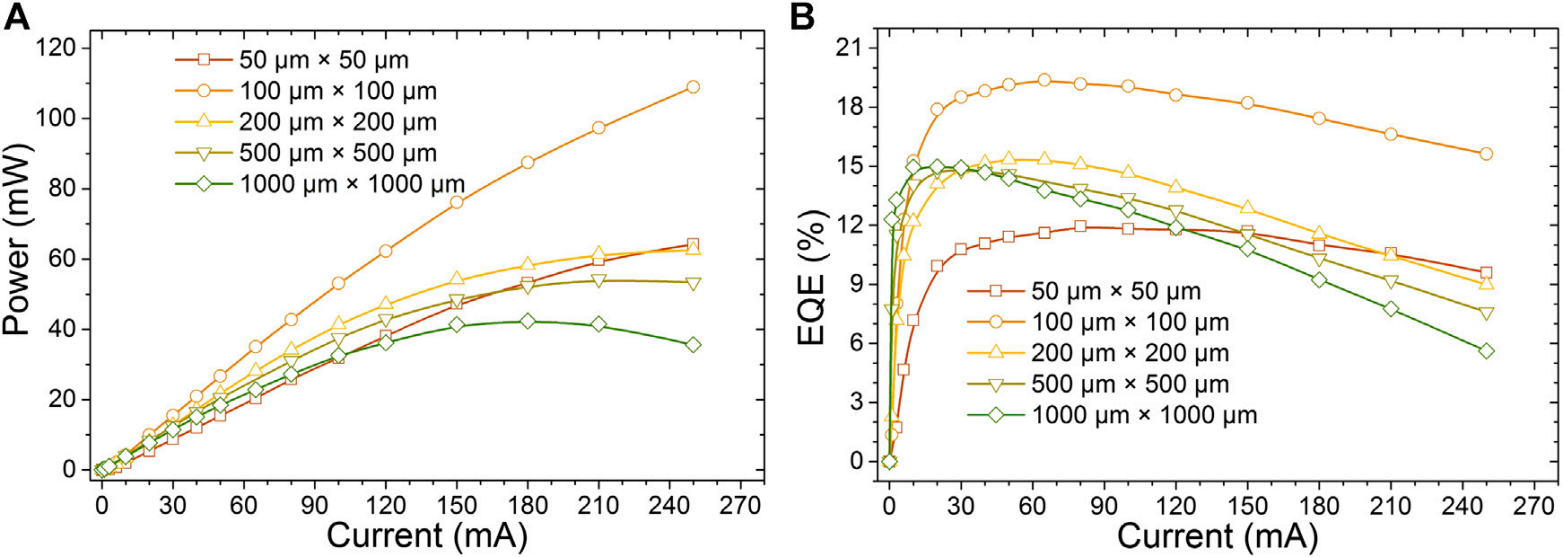
**(A)** Optical power versus electrical current and **(B)** the EQE as a function of the current of PG micro-LEDs in different sizes.

The optical output power and the external quantum efficiency (EQE) for these PG micro-LEDs of different sizes are presented in Figures 2A,B, which show that the optical performance is enhanced as the device size decreases from 1,000 μm × 1,000 µm–100 μm × 100 µm as a general trend. Higher optical output power can be obtained in smaller devices at a given current level. However, the 50 μm × 50 µm device exhibits a smaller optical output power and EQE than others at the lower current density. As the 50 μm × 50 µm device has the smallest ratio of active lighting area, which may be one of the underlying reasons. Nevertheless, it seems that the small ratio of active lighting area is not the only reason for such a degradation for the 50 μm × 50 µm device. To figure out the underlying reasons of these observations, a series of characterization methods were performed and the results were analyzed.

**FIGURE 3:**
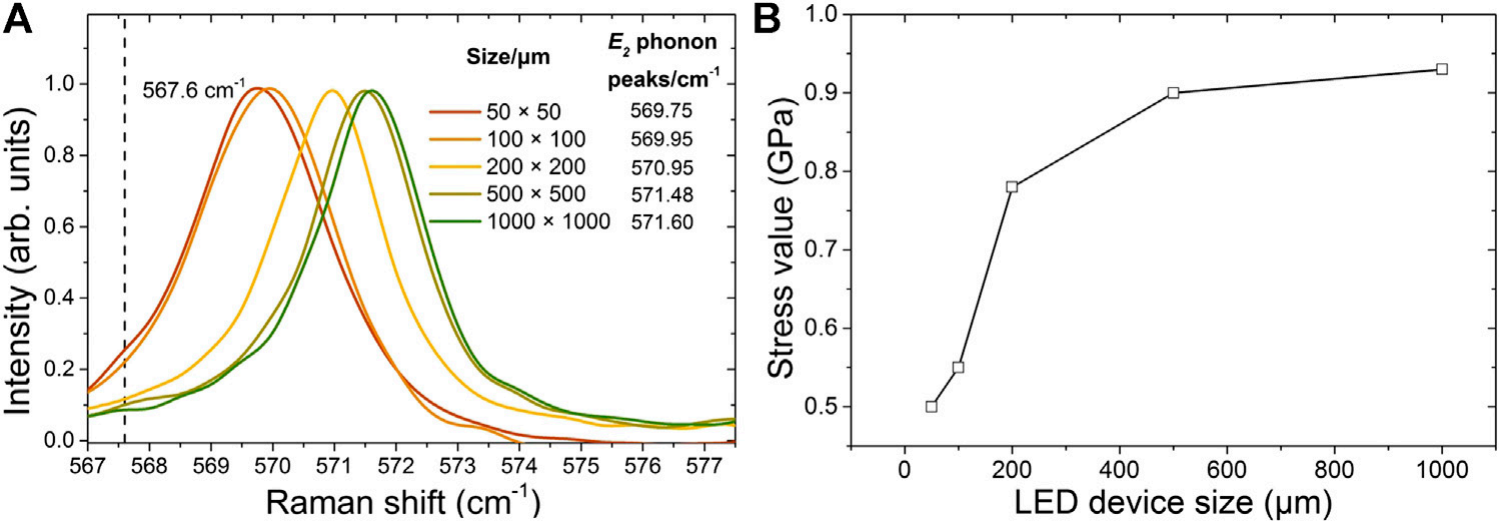
**(A)** Raman spectra of *E*_*2*_ (high) phonon peaks for the PG micro-LEDs of different sizes; **(B)** the in-plane compressive stress of different sized PG micro-LEDs.

Raman scattering is a widely used method to study the strain for III-nitrides (Tian et al., 2015). As the *E*_*2*_ (high) phonon frequency is sensitive to strain, it has been extensively applied to quantify the in-plane stress in GaN (Davydov et al., 1997; Tripathy et al., 1999). It is accepted that the peak of *E*_*2*_ phonon in the unstrained GaN layer is 567.6 cm^−1^ (Davydov et al., 1998), and the blueshift for this phonon frequency indicates the in-plane compressive stress, whereas a redshift indicates the in-plane tensile stress. As depicted in Figure 3A, Raman spectra of *E*_*2*_ phonon peaks for different PG micro-LEDs show that all the measured phonon frequencies of the PG micro-LEDs are blueshifted, which accounts for the in-plane compressive stress. From Figure 3A, we can also see that with the size decreasing from 1,000 μm × 1,000 µm–50 μm × 50 μm, the *E*_*2*_ phonon peaks move toward to the unstrained 567.6 cm^−1^, which indicates that the in-plane compressive stress is released with the size decreasing. To facilitate the analysis, the in-plane compressive stress was further calculated according to σ=(Δω/4.3) cm⋅GPa (Tripathy et al., 2002), where *σ* is the biaxial stress and Δ*ω* is the *E*_*2*_ phonon peak difference between the strained GaN and unstrained GaN (567.6 cm^−1^). The calculated in-plane compressive stress values of different sized PG micro-LEDs are presented in Figure 3B. From Figure 3B, it is clear that the in-plane stress reduces as the size decreases. This is because at the edge of micro-LEDs, the growth is free and nearly no in-plane stress. From the edge to the center of the micro-LEDs, the stress becomes large. Smaller sized micro-LEDs have more edge area, so it shows large area ratio for strain relaxation. The reduced in-plane compressive stress for the GaN template layer grown on the sapphire substrate correspondingly suppresses the QCSE level in the InGaN/GaN quantum wells. The reduced QCSE suggests that by decreasing the PG micro-LED size the output power and EQE can be improved. However, as can be observed from Figure 2, when the chip size is below 100 μm × 100 μm, the other effects need to be taken into account because the device performance decreases.

**FIGURE 4:**
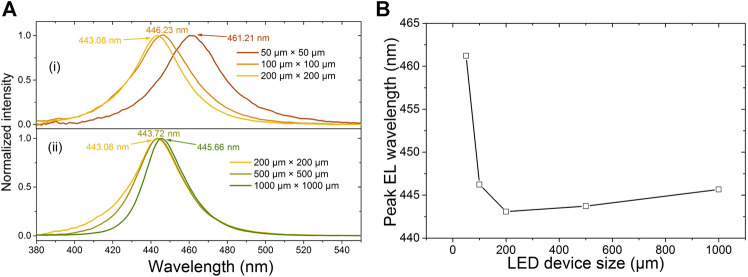
EL spectra of the PG micro-LEDs of different sizes at 20 mA: **(A)** 1,000 μm × 1,000 μm, 500 μm × 500 μm, and 200 μm × 200 µm devices; and **(B)** 200 μm × 200 μm, 100 μm × 100 μm, and 50 μm × 50 µm devices. **(C)** Peak EL wavelength of different sized PG micro-LEDs.

As the in-plane stress reduces, the blueshift for the electroluminescence wavelength caused by the reduced QCSE in the quantum wells is expected. The EL spectra and the peak EL wavelength for the studied PG micro-LEDs at 20 mA are depicted in Figure 4. As expected, the emission wavelength is blueshifted with the size decreasing from 1,000 μm × 1,000 µm–200 μm × 200 μm, as can be seen from Figures 4Aii,B; however, the blueshift for the wavelength is very small. This phenomenon is because the blueshift for the wavelength caused by the reduced QCSE in the quantum wells can be partially compensated by the redshift, which is due to the more indium incorporation into the quantum wells with decreased stress (Johnson et al., 2004; Ju et al., 2012). On the other hand, the wavelength shows redshift as the size is decreased from 200 μm × 200 µm–50 μm × 50 μm, as can be seen from Figures 4Ai,B, which is not expected. For the 50 μm × 50 µm and 100 μm × 100 µm cases, with the in-plane stress further reducing, the effect of the indium incorporation is stronger than that of the QCSE suppression, so we see the wavelength redshift. However, this high concentration of indium not only makes the peak wavelength redshifted, but also introduces more defects (Johnson et al., 2004), which can be proved by the shift of EQE peak toward higher current (Zhang et al., 2015), as shown in Figure 2B. It has also been reported that point defects enhance the nonradiative recombination (Cao et al., 2003), which can be another reason for the lower output power and EQE for the 50 μm × 50 µm size when the current is smaller than 120 mA.

**FIGURE 5:**
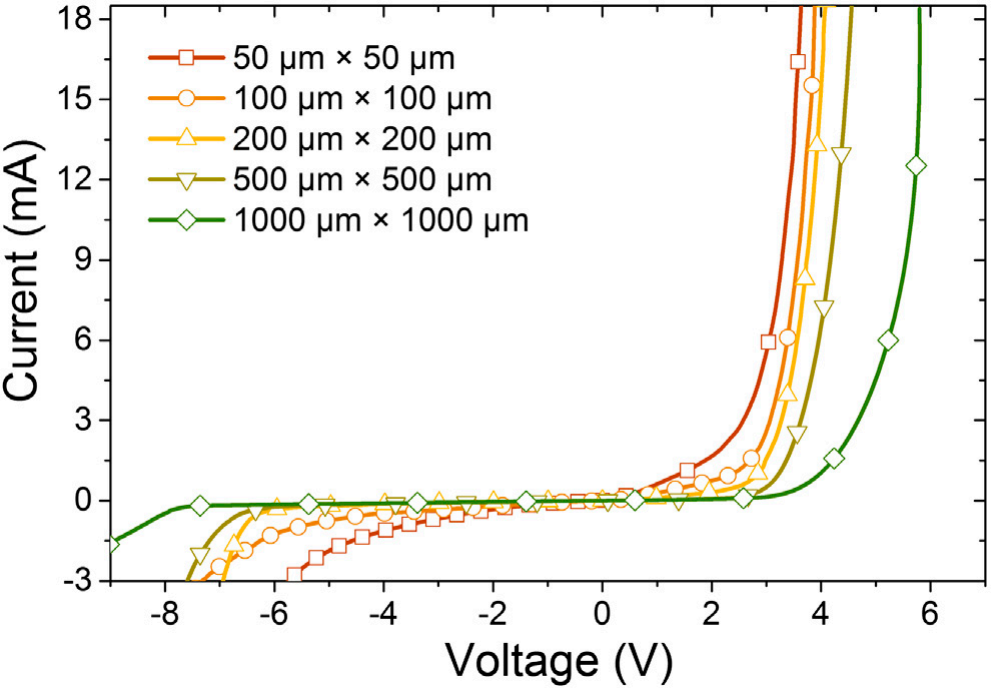
I–V characteristics for PG micro-LEDs of different sizes.

We further carried out the current voltage (I–V) characteristics for the PG micro-LEDs of different sizes in Figure 5. From Figure 5 we can see that the reverse leakage current increases when the PG micro-LED size is decreased, indicating more defects in the quantum wells (Cao et al., 2003). It is worth noting that the 50 μm × 50 µm device shows the largest leakage current when the device is reversely biased, which is a signature of the high defect density. In our case, the higher defect density in the quantum wells is ascribed to the higher indium incorporation efficiency for the smaller PG micro-LEDs, and therefore, further epi-growth optimizations are required.

## Conclusion

In this work, to study the size effect on strain release, strain-reduced micro-LEDs in 50 μm × 50 μm, 100 μm × 100 μm, 200 μm × 200 μm, 500 μm × 500 μm, and 1,000 μm × 1,000 µm sizes were grown on patterned c-plane sapphire substrates by the MOCVD technique. Various characterization methods were performed to study the strain for different sizes. The output power and EQE characteristics show that the optical performance can be enhanced when the PG micro-LED size is reduced from 1,000 μm × 1,000 µm–100 μm × 100 µm. The improved EQE is due to the reduced QCSE in the InGaN/GaN quantum wells for those PG micro-LEDs. However, for the 50 μm × 50 µm size, the optical performance is limited by the smallest effective lighting area and additional defects induced by higher indium incorporation. Therefore, in our case, the 100 μm × 100 µm size delivers the highest output power and EQE. In summary, our experimental results indicate that to obtain high-quality epitaxy based on partitioned growth, a small size is needed provided that the defects density and margin ratio are carefully controlled. Since the partitioned growth method is easy and low-cost to apply to the sapphire substrate LEDs growth process, such directly grown micro-LEDs hold great promise for being adopted in commercial product lines.

## Data Availability

The original contributions presented in the study are included in the article/Supplementary Material, further inquiries can be directed to the corresponding authors.
